# PEAKS AND VALLEYS: BIPOLAR DISORDER, RAPID CYCLERS AND ENERGY DRINKS CONSUMPTION

**DOI:** 10.1192/j.eurpsy.2023.1470

**Published:** 2023-07-19

**Authors:** M. Calvo Valcárcel, M. A. Andreo Vidal, P. Martinez Gimeno, P. Pando Fernández, B. Rodriguez Rodriguez, N. Navarro Barriga, M. Fernández Lozano, M. J. Mateos Sexmero, M. D. C. Vallecillo Adame, T. Jimenez Aparicio, C. de Andres Lobo, M. Queipo de Llano de la Viuda, A. A. Gonzaga Ramirez, G. Guerra Valera

**Affiliations:** PSYCHIATRY, Hospital Clínico Universitario de Valladolid, VALLADOLID, Spain

## Abstract

**Introduction:**

Bipolar Disorder (BD) is considered a serious mental disorder characterized by a changing mood that fluctuates between two completely opposite poles. It causes pathological and recurrent mood swings, alternating periods of exaltation and grandiosity with periods of depression. We talk about rapid cyclers when four or more manic, hypomanic or depressive episodes have occurred within a twelve-month period. Mood swings can appear rapidly. Approximately half of the people with bipolar disorder may develop rapid cycling at some point.

**Objectives:**

Presentation of a clinical case about a patient with Bipolar Disorder with rapid cycling and poor response to treatment.

**Methods:**

Review of the scientific literature based on a clinical case.

**Results:**

33-year-old male, single, living with his mother, under follow-up by mental health team since 2012. First debut of manic episode in 2010. The patient has filed multiple decompensations related to consumption of toxics (alcohol and cannabis). Currently unemployed. He attended to the emergency service in June 2022 accompanied by his mother, who reported that he was restless. The patient refers that he has interrupted the treatment during the vacations, having sleep rhythm disorder with abuse of caffeine drinks. Currently the patient does not recognize any consumption.The patient reports that during the village festivals he felt very energetic, occasionally consuming drinks rich in taurine and sugars, even having conflicts with people of the village. Finally, the patient was stabilized with Lithium 400 mg and Olanzapine. In September, the patient returned to the emergency service on the recommendation of his referral psychiatrist due to therapeutic failure. The only relevant finding we observed in the analytical determinations were low lithium levels (0.4 mEq/L). The transgression of sleep rhythms and the abuse of psychoactive substances required the admission of the patient to optimize the treatment (Clozapine, Lithium, Valproic Acid). At discharge, he is euthymic, has not presented behavioral alterations and is resting well. Finally, it was decided that the patient should go to the Convalescent Center to continue treatment and achieve psychopathological stability.

**Conclusions:**

Bipolar disorder is an important mental illness, having an incidence of 1.2%, being responsible for 20% of all mood disorders. Therefore, it is important to perform an adequate and individualized follow-up of each patient. Treatment with mood stabilizers tries to improve and prevent manic and depressive episodes, improving chronicity and trying to make the long-term evolution as good as possible, being important psychoeducation and psychotherapy.

**Disclosure of Interest:**

None Declared

